# Cell Therapy for Critical Limb Ischemia: Advantages, Limitations, and New Perspectives for Treatment of Patients with Critical Diabetic Vasculopathy

**DOI:** 10.1007/s11892-021-01378-4

**Published:** 2021-03-02

**Authors:** Y. Gu, A. Rampin, V. V. Alvino, G. Spinetti, P. Madeddu

**Affiliations:** 1 Bristol Medical School, Translational Health Sciences, University of Bristol, Upper Maudlin Street, Bristol, BS2 8HW UK; 2 Laboratory of Cardiovascular Research, IRCCS, MultiMedica, Milan, Italy

**Keywords:** Critical Limb Ischemia, Diabetes Mellitus, MicroRNAs, Microvesicles, Proangiogenic cells, Vascular Stem and Progenitor cells

## Abstract

**Purpose of Review:**

To provide a highlight of the current state of cell therapy for the treatment of critical limb ischemia in patients with diabetes.

**Recent Findings:**

The global incidence of diabetes is constantly growing with consequent challenges for healthcare systems worldwide. In the UK only, NHS costs attributed to diabetic complications, such as peripheral vascular disease, amputation, blindness, renal failure, and stroke, average £10 billion each year, with cost pressure being estimated to get worse. Although giant leaps forward have been registered in the scope of early diagnosis and optimal glycaemic control, an effective treatment for critical limb ischemia is still lacking. The present review aims to provide an update of the ongoing work in the field of regenerative medicine. Recent advancements but also limitations imposed by diabetes on the potential of the approach are addressed. In particular, the review focuses on the perturbation of non-coding RNA networks in progenitor cells and the possibility of using emerging knowledge on molecular mechanisms to design refined protocols for personalized therapy.

**Summary:**

The field of cell therapy showed rapid progress but has limitations. Significant advances are foreseen in the upcoming years thanks to a better understanding of molecular bottlenecks associated with the metabolic disorders.

## Introduction

Type 2 diabetes (T2D) and its complications have become one of the biggest threats to global health and economy [1]. Approximately 9% of adults in the UK were affected by diabetes in 2015 and 90% of them were diagnosed with T2D [2]. Endothelial dysfunction (ED) is acknowledged as a central player in diabetic vascular complications, including retinopathy, cardiomyopathy, renal failure, and diabetic foot [3]. The evolution of diabetes-related peripheral vascular disease (PVD) has been described at the cellular and tissue level. Insulin resistance and glucose level instability/hyperglycaemia are key causative factors in the onset of microvascular ED and microangiopathy, which worsens the ischemic effects of concurrent atherosclerotic macroangiopathy. This detrimental combination results in progressive deterioration of tissue perfusion ultimately leading to critical limb ischemia (CLI) [4, 5].

CLI is defined as a clinical syndrome of chronic ischemic pain, skin ulcerations, and eventually gangrene, and it is associated with a highly increased mortality risk. Current treatments encompass pharmacological therapy and revascularization. However, a significant number of CLI patients are ineligible to reperfusion treatments because of the anatomic location of the lesions, the extent of the disease, and comorbidities. Such “no option CLI” (NO-CLI) patients often require limb amputation as an extreme life-saving procedure [6, 7]. However, the prognosis remains poor; diabetes-related amputations have a high risk of mortality, with a 5-year survival rate of 40–48%.

In the last 20 years, regenerative medicine treatments have been proposed to address the unmet clinical needs of CLI patients. Chronologically, gene therapy using growth factors (GFs) was first introduced with the objective of fuelling reparative angiogenesis in affected muscles. The approach has shown some encouraging results, but continues to bear important limitations, which were mainly attributed to the poor gene transfer yield and limited duration of the effect, as well the inability of a single growth factor to resolve the pathogenic complexity of underlying molecular mechanisms [8–10]. More recently, the attention has shifted on micro-RNAs (miRNAs). Several short non-coding RNA (ncRNA) sequences reportedly contribute to the pathogenesis and progression of CLI through modulation of several downstream genes [11–13]. Moreover, miRNAs form functional clusters, which cooperate but also interfere with each other in different pathophysiological conditions. Hence, great interest has been focused on miRNA therapeutics and on miRNA regulating drugs [14–16]. Other categories of ncRNAs, including long ncRNA (lncRNAs) and circular RNAs (circRNA), are emerging as key players in diabetes cardiovascular complications [17, 18]. Finally, cell therapy has also been employed as a regenerative treatment, with a growing number of clinical trials exploring the potential in ischemic disease and CLI [7]. The benefit of injecting cells in ischemic tissues is mainly attributed to the regulated release of growth factors, cytokines, and genetic material, either in soluble or vesicle-embedded form. Therefore, cell therapy represents a more global method to address the pathophysiological aspects of vascular disease.

This review highlights the current state of cell therapy for the treatment of CLI. First, we provide some examples of studies using different cell types; second, we indicate how T2D can influence the cell phenotype and behaviour, potentially limiting the benefits or causing undesired effects; and third, we will discuss the contribution of ncRNAs in the positive and adverse responses to the cell-based therapies. Our perspective is that, in the near future, the fields of cell therapy, gene therapy, and ncRNA therapy may advantageously converge into an all-inclusive approach capable of improving the quality of life and outcome of patients suffering from ischemic complications of diabetes.

## Cell Therapy for Vascular Regeneration in Critical Limb Ischemia

Recent meta-analyses conducted on a multitude of clinical trials suggest that autologous stem cell-based therapies can actually improve the clinical outcome of diabetes-related CLI patients [19, 20]. However, some level of inconsistency has emerged; highlighting the need for further studies to better define all the critical aspects of the therapeutic protocol, including the source of the optimal cell type, the identification and screening for predictors of efficacy, as well as the administration modalities such as injection sites and treatment frequency. Table 1 illustrates examples of candidate cell types, classified according to the tissue source.

**Table 1:** Human stem and progenitor cell sources and types. Table source adapted in modified version from Hmadcha et al [21], and Soria-Juan et al [22]. *AF*, amniotic fluid; *AF-MSCs*, amniotic fluid-derived mesenchymal stem cells; *AT*, adipose tissue; *AT-MSCs*, adipose tissue-derived mesenchymal stem cells; *BM*, bone marrow; *BM-MSCs*, bone marrow-derived mesenchymal stem cells; *BM-MNCs*, bone marrow-derived mononuclear cells; *EPCs*, endothelial progenitor cells; *HSCs*, haematopoietic stem cells; *PB*, peripheral blood; *PB-MSCs*, peripheral blood-derived mesenchymal stem cells; *PB-MNCs*, peripheral blood-derived mononuclear cells; PL, placenta; *PL-MSCs*, placenta-derived mesenchymal stem cells; *UC*, umbilical cord; *UC-MSCs*, umbilical cord-derived mesenchymal stem cells; *WJ*, Wharton’s jelly; *WJ-MSCs*, Wharton’s jelly-derived mesenchymal stem cells

Sources	Tissue	Stem and progenitor cells
Adult tissues	BM	HSCs, EPCs, BM-MNCs [23, 24]
	PB	PB-MNCs [25]
	BM stroma	BM-MSCs [26]
	PB stroma	PB-MSC [27]
	AT	AT-MSCs [28]
	Skeletal and cardiac muscle, skin, cartilage, oral cavity, dental pulp, etc.	MSCs and pericytes [29, 30]
Extraembryonic/neonatal tissues	UC	UC-MSCs [31]
	WJ	WJ-MSCs [32]
	AF	AF-MSCs [33]
	PL	PL-MSCs [34]

In the next sections, we summarize the latest results in the quest for a suitable cell therapy approach for NO-CLI. Clinically relevant developments in the field have been assessed by conducting a PubMed search of articles published in the last 5 years, extending then to essential prior publications that have led to current state of the art. In particular, we have revised the available evidence on mesenchymal stromal cells, mononuclear cells, and resident vascular progenitor cells.

### Mesenchymal Stromal Cells

Mesenchymal stromal cells (MSCs) represent the leading cell type used in regenerative therapies for the treatment of autoimmune, inflammatory, and vascular diseases. MSCs secrete a wide variety of pro-inflammatory and anti-inflammatory cytokines, GFs, and prostaglandins under resting and inflammatory conditions [21, 22, 35, 36]. These molecules are associated with immunomodulation, anti-apoptosis, angiogenesis, cell growth and differentiation, as well as anti-fibrosis and chemo-attraction [22, 37]. MSCs are currently considered among the most promising cell types as a regenerative option to the surgical treatment of CLI, as they provide some peculiar advantages [38]. Additionally, they reportedly express low levels of mayor histocompatibility complex (MHC) class II molecule, allowing for their use in allogenic treatments that would overcome specific functional limitations typical of T2D [39]. MSCs of different origins have been pursued as candidate cell types for cell therapy of NO-CLI patients [40•], with ongoing clinical trials mainly focusing on bone marrow-derived MSCs (BM-MSCs).

CHAMP (Clinical and Histologic Analysis of Mesenchymal stromal cells in amPutations, trial number: NCT02685098) is an open label, single centre, non-randomized phase I clinical trial started in September 2017 and due to be completed by the year 2025. The study plans to enrol 16 patients requiring semi-elective lower extremity major amputation and aims to verify the safety and efficiency of concentrated BM aspirate (cBMA) and BM-MSCs intramuscular injection to NO-CLI patients [38]. This study was set up to better understand the mechanisms involved in a previous phase I study showing that intramuscular administration of MSCs-containing cBMA resulted in a 1- and 5-year amputation-free survival (AFS) rate of 86 and 74% respectively, a result comparable to that achieved through revascularization [41]. Another endpoint of the trial is to determine the safety and efficacy at 6 months after injection, but also to inquire the short-term engraftment of BM-MSCs, their remodelling and paracrine activities after injection.

SAIL (allogeneic mesenchymal stromal cells for angiogenesis and neovascularization in no-option ischemic limbs, trial number: NCT03042572) is a randomized, double-blind, placebo-controlled clinical trial begun in 2018 and expected to end by July 2021. It will provide additional data on the safety and potential efficacy of allogeneic BM-MScs treatment for NO-CLI [42].

### Mononuclear Cells

Following the isolation of CD34^+^ angiogenic mononuclear cells (MNCs) in 1997, a large number of studies have been dedicated to the evaluation of efficacy and safety as angiogenic cell therapy for NO-CLI patients [43••]. In a pioneering study, Tateyshi and colleagues demonstrated that intramuscular administration of BM-MNCs improved transcutaneous oxygen pressure (TcPO2), rest pain, and walking distance after 4-week and 6-month-follow ups compared to peripheral blood-derived MNCs (PB-MNCs), which were used a cellular control [44]. Several other studies reported a beneficial effect of autologous BM-MNCs and cBMA on AFS rate, amputation, pain, quality of life, Rutherford classification, and ankle brachial index (ABI) [45–47]. The benefit was attributed to the support of neovascularization within the ischemic tissues [35, 48•], via direct engraftment, differentiation into appropriate tissue cells, and paracrine interaction with and stimulation of resident vascular cells [49]. A novel paracrine mechanism has been recently described for BM-MNCs, involving downregulation of the endogenous nitric oxide synthase (NOS)-inhibitor asymmetric dymetylalanine [50].

Both BM-MNCs and PB-MNCs are still under preclinical and clinical investigation [51–54]. The derivation of MNCs from PB is attractive considering the procedural risk associated with BM extraction [55]. Nonetheless, different clinical trials reported contradictory outcomes with regard to the improvement of ABI, ulcer healing, pain-free walking time, and limb salvage following the infusion of MNCs in CLI patients [22, 24, 25, 35, 37]. The reason for such variability might reside in the heterogeneous nature of MNCs. Therefore, experts in the field have moved to the evaluation of better defined MNC sub-populations, such as CD34^+^ and CD133^+^ MNCs. The latest meta-analysis conducted on clinical trials involving CD34^+^ MNCs indicates these cells can effectively reduce the rate of major amputation, while improving ulcer healing parameters [56]. Another concept emerging from meta-analysis is that the response to CD34^+^ MNCs may be dose-dependent. In support to this hypothesis, treatment of NO-CLI with GCS-F-mobilized- or enriched CD34^+^ MNCs displayed encouraging results [51, 57, 58]. Regarding mechanisms, previous studies on CD34^+^ MNCs showed that they can improve limb reperfusion through extracellular vesicle (EV)-mediated delivery of angiogenic miRNAs [22, 48•, 59, 60•]. In a diabetic mice model of CLI, the use of conditioned media collected from human CD133^+^ subset of CD34^+^ MNCs accelerated wound closure and reparative angiogenesis through paracrine signalling [61•]. Accordingly, a small-sized clinical study on the CD133^+^ subset of granulocytes colony-stimulating factor (GCS-F)-mobilized CD34^+^ PB-MNCs also provided encouraging results (75% general improvement, 62.5% complete wound healing) [62].

Recent data on the application of specific MNCs subpopulations to NO-CLI treatment are therefore promising. Future studies will necessarily benefit from the deeper knowledge of the molecular mechanisms specific to T2D-related CLI.

### Resident Vascular Progenitor Cells

Damage and pauperization of vascular cells, namely endothelial cells (ECs), vascular smooth muscle cells (VSMCs), and pericytes, represent a hallmark of microangiopathy. Therefore, supplying a boost of vascular progenitor cells to the site of ischemic injury, as a method of therapeutic vasculogenesis, could alleviate local quantitative and qualitative deficits and promote tissue reperfusion and repair.

Pericytes represent a viable source for such an endeavour, due to their key role in supporting a stable vascularization and in acting as the interface between vasculogenesis and myogenesis [63••]. Recent evidence indicates in fact that pericytes not only play a role in vascular reparative processes, but also in regeneration of myocytes together with satellite cells [63••]. Preclinical evidence suggests that transplantation of pericytes could exert positive effects in different pathologies, including myopathies and ischemic disease [64••, 65–67]. The use of pericytes as a cell source for the treatment of NO-CLI is only recently beginning to gather attention. Our group has provided compelling preclinical evidence of efficacy and safety in murine models [29, 61, 68•, 69, 70]. Nonetheless, autologous pericytes may be affected by underpinning pathology, as observed in T2D. We reported that pericytes from limb muscles of patients with diabetic vasculopathy display elevated levels of oxidative stress causing a dysfunctional phenotype [64••]. In T2D mice, treatment with dimethyl-2-oxoglutarate (DM-2OG), a tricarboxylic acid cycle metabolite with antioxidant properties, could restore pericyte redox balance and mitochondrial function, while concurrently allowing for enhanced pericyte-endothelial crosstalk [71•]. These results suggest that metabolic therapy may help to prevent or slow down vasculopathy in skeletal muscles of people with diabetes. Alternatively, allogeneic approaches could be considered. A study conducted in mice of induced limb ischemia showed an improvement in blood flow and collateral artery diameter recovery in animals treated with allogeneic pericytes [72].

Regarding other vascular cells, a recent small-size phase Ib clinical trial evaluated the efficiency of a 1:1 formulation containing ECs genetically modified to constitutively express angiopoietin-1 (ANG1) and VSMCs transfected with a vascular endothelial growth factor (VEGF) retroviral vector for the treatment of NO-CLI. Some improvements were observed. One-year amputation-free survival rate was 72% (13/18) for Rutherford 4 and 5 patients, whereas all 5 patients with Rutherford 6 underwent amputation. Of the 12 with non-healing ulcers, 6 had complete healing and 2 others had a significant reduction (> 66%) in ulcer size. Outcomes did not associate with dose stratification. No severe adverse events were observed related to the therapy [73].

Another promising approach that is expected to reach the clinical trial stage soon involves the application of human-induced pluripotent stem cells- (hiPSCs) or SCs-derived EC products [74–77]. Evidence of efficacy was provided by a recent preclinical study that reported an accelerated reperfusion and increased capillary density in CLI mice treated with a clinical grade hSCs-derived EC product [78]. Interestingly, such product was composed of a heterogeneous cell population, enriched in CD144 and CD31-positive-differentiated ECs, but also expressing mesenchymal and pericyte markers, thus indicating both ECs and pericytes could participate in the therapeutic effect. Altogether, these studies suggest that combinations of specialized vascular cells may be optimally suited to regenerate the vasculature of NO-CLI patients.

## Diabetes-Related Limitations

**Fig. 1:**
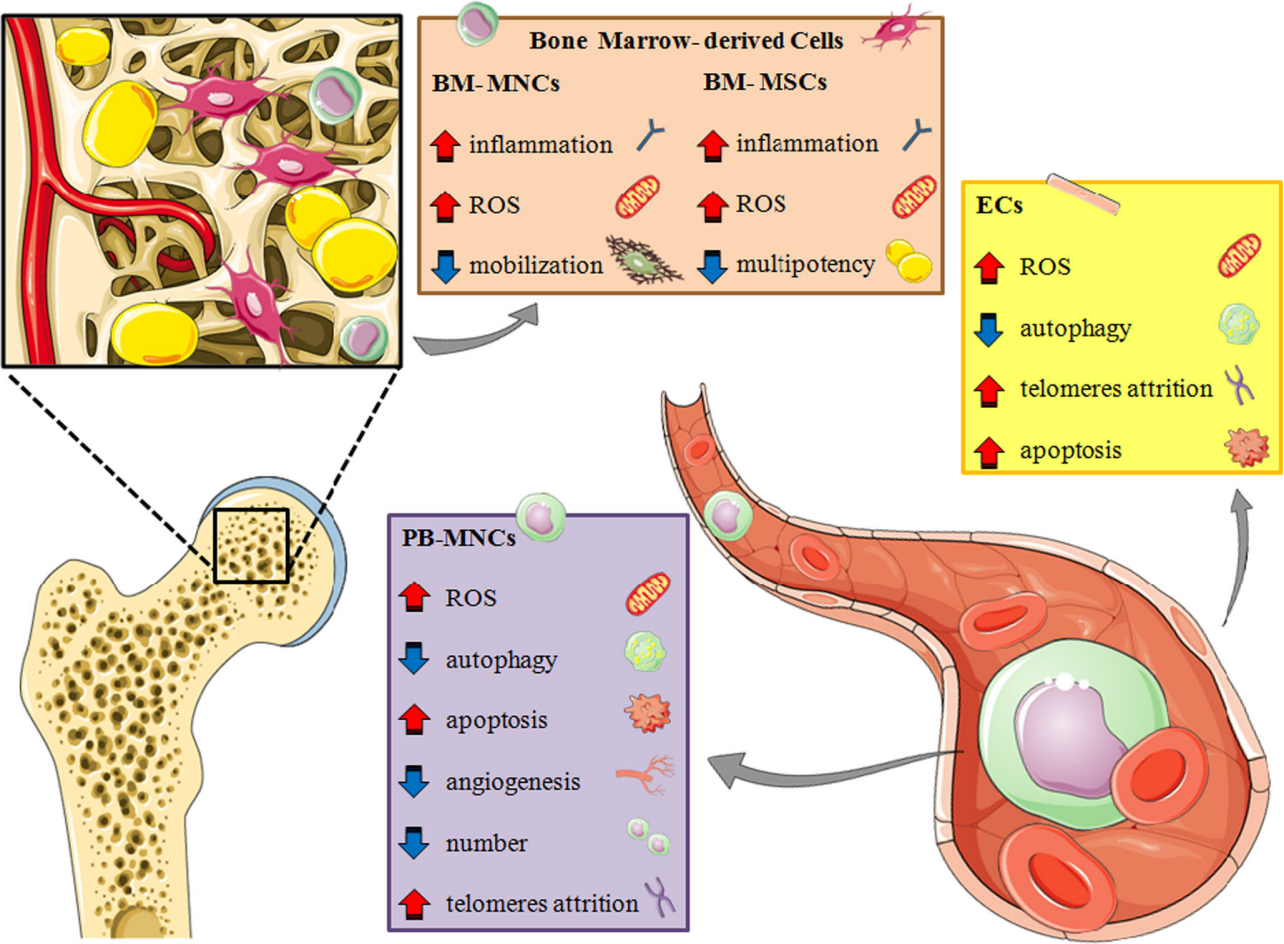
Damaging effect of diabetes on stem/progenitor cells in bone marrow and peripheral circulation. BM bone marrow, MNCs mononuclear cells, MSCs mesenchymal stromal cells, ECs endothelial cells, PB peripheral blood. This figure was created using images from Servier Medical Art Commons Attribution 3.0 Unported License. (http://smart.servier.com). Servier Medical Art by Servier is licensed under a Creative Commons Attribution 3.0 Unported License

The harsh tissue environment created by the combination of diabetes and ischemia can hamper the functionality of candidate cell products for autologous and, potentially, allogeneic treatments (Fig. 1). The following sections summarize relevant molecular targets to improve cell therapy outcomes in patients with T2D.

### Molecular Alterations in Ischemic Tissue and Circulating and Bone Marrow Resident Progenitor Cells

T2D is associated with increased levels of mitochondrial and endoplasmic reticulum (ER) oxidative stress, resulting from hyperglycaemia-induced activation of the electron transfer chain (ETC) in the mitochondria [79]. Consequently, large amounts of reactive oxygen species (ROS) are produced and diffused to the ER through the mitochondria-associated membranes (MAMs).

An impairment of the protein-folding machinery triggers autophagy, an important pathway that allows cells to dispose of the ROS-damaged macromolecules. However, autophagy is inhibited by insulin through the activation of mammalian target of rapamycin (mTOR), which results in unc-51 like autophagy activating kinase 1 (ULK1) inactivation under insulin resistance conditions [80]. In line with this, recent evidence reports an inhibition of mitophagy (mitochondria-specific autophagy) in PB-MNCs from patients with T2D [81]. Moreover, tumour suppressor-programmed cell death-4 (*PDCD4*) overexpression was demonstrated in diabetic PB-MNCs [82•]. These results mirror the recent observation that the inhibition of autophagy associates with an increased expression of *PDCD4* in a human cancer cell line [83].

Importantly, hyperglycaemia-induced accumulation of advanced glycation end-products (AGEs) and ROS production concurrently results in endothelial nitric oxide synthase (eNOS) dimer uncoupling, which leads to the accumulation of highly reactive nitrogen species [84–86]. This, in turn, affects the mobilization and function of BM-derived progenitors, which are thought to have pro-angiogenic and healing potential. Selective inhibition of the PI3K/Akt signalling pathway during insulin resistance can contribute to eNOS-reduced expression and function, thereby expanding the effect of diabetes on NO equilibrium [87, 88]. Excess ROS production accelerates telomere length shortening, or telomeres attrition, a process that is exacerbated in T2D. Telomeres attrition is significantly higher in ECs and PB-MNCs from patients with T2D, and is associated with faster vascular ageing [89].

Patients with T2D exhibit decreased circulating CD34^+^ MNCs, a deficit that predicts the risk of complications and death [90, 91, 92••, 93, 94]. Various mobilizing mechanisms contributing to the liberation of BM-resident progenitor cells in the circulation are profoundly altered in T2D, a defect known as mobilopathy or myelokathexis, with negative consequences for physiologic hematopoiesis, immune regulation, and tissue regeneration, as reviewed in [95]. Nociceptive perception is depressed in CLI patients favouring the formation of foot ulcers. Interestingly, depression of nociceptive reflexes can also contribute to mobilopathy in mice and patients with complicated T2D, reducing the recruitment of reparative cells that express the nociceptive receptors [96, 97].

In addition to causing an altered progenitor cell mobilization, T2D was shown to drastically alter the cellular composition of BM in favour of adipose tissue accumulation; this transformation can hinder the intrinsic regenerative potential of resident mesenchymal and hematopoietic cells and compromise their utility for therapeutic transplantation purposes [98]. Moreover, our group has discovered that the in vitro migratory activity of CD34^+^ MNCs positively correlated with cardiovascular death, independently from other predictive factors, such as age, coronary artery disease, serum C-reactive protein, and glomerular filtration rate [82•]. At a first glimpse, these findings may appear counterintuitive, considering that migration is instrumental to BM-derived cells to reach damaged tissues, unless these cells have turned into deleterious elements because of T2D. In line with this possibility, a pilot, phase IIa, double-blind, randomized, placebo-controlled trial (NCT02790957) conducted in patients with T2D with ischemic wounds showed that mobilization treatment with the CXCR4 antagonist plerixafor had negative effects on wound healing as compared to the placebo, leading to premature trial termination after a pre-planned interim analysis [99].

Equilibrium in the paracrine- and GAP junction-supported crosstalk between vascular mural cells is impaired in T2D-related CLI. Evidence suggests that such damage includes permanent as well as transient epigenetic modifications that could hinder the efficacy of pericytes and ECs in autologous cell therapy [72, 100, 101]. Interestingly, the pericyte-EC crosstalk is also altered in BM, with depression of AKT-dependent angiocrine signalling being a hallmark of local microangiopathy [102, 103]. Transfection with the adenoviral vector carrying the coding sequence for constitutively active AKT rescued functional defects and angiocrine signalling in BM pericytes, suggesting this could be a molecular target to restore the progenitor cell niche [102]. Likewise, targeting inflammatory cytokines could improve BM function in T2D. Pharmacological inhibition of MCP-1 signalling reduced adiposity in BM of T2D mice, restoring, at least in part, the balance between adipogenesis and hematopoiesis [104].

Taken together, these observations indicate that T2D significantly affects the functionality of virtually all the cell types currently considered as viable candidates for CLI cell therapy. Therefore, innovative techniques able to restore the functionality of the transplanted cells, as well as the development of reliable allogeneic alternatives, could represent valuable choices for future research.

### MicroRNAs in Diabetes-Related Vascular Disease: Implications for Cell Therapy

The prolonged modification of the cellular epigenetic profile because of a metabolic insult is known as metabolic memory. Epigenetic modifications could significantly limit the efficacy of autologous cell therapy in CLI-T2D patients [105–108]. Importantly, miRNAs can affect the epigenetic mechanisms by targeting key enzymes involved in establishing epigenetic memory. For instance, T2D is associated with upregulation of pro-inflammatory miRNAs (e.g., miR-125b, mir-146a-5p, and miR-29a-3p) in aortic ECs, causing a chronic inflammatory status that closely resembles the senescence-activated secretory phenotype (SASP). Notably, this phenomenon is reversible, with miRNA inhibition being able to reverse NF-κB pathway activation in vascular cells [109]).

The miR-126 offers another relevant example of the miRNAs ability to modulate metabolic memory at different levels. Indeed, miR-126 can bind the 3′-UTR regions of, among others, insulin receptor substrate-1 (IRS-1), phosphatidyl inositol 3-phosphate kinase receptor-2 (PI3KR-2), sprouty related EVH1 domain-containing-1 (SPRED-1), and disintegrin and metalloproteinase domain-containing protein-9 (ADAM-9). This multiple targeting respectively results in IR modulation, EC survival and proliferation, and modulation of cell migration [110–113]. In T2D, miR-126 is reportedly downregulated, possibly due to a compensatory response to insulin resistance, thereby contributing to ED and vascular disease [114]. In diabetic mice, restoration of miR-126 levels reduced PVD development [14, 113]. MNCs isolated from PB of diabetic patients displayed a downregulation of miR-126, resulting in reduced proliferation and increased apoptosis through upregulation of its target Spred1; whereas, forced miR-126 expression improved efferocytosis in human macrophages [111].

Overexpression of circulating miR-15a and miR-16-1, which shares a common pre-pri-miRNA under the transcriptional control of E2F-1, is predictive of restenosis and limb amputation in T2D-CLI patients undergoing percutaneous angioplasty [115••]. In line with a negative influence of these microRNAs on cell therapy, transplantation of PB-MNCs ex vivo-engineered with anti-miR-15a/16 improved post-ischemic blood flow recovery and muscular arteriole density in immunodeficient mice [115••].

In vitro studies unravelled the role of hypoxia in the modulation of miR-532-5p and miR-210 in pericytes and MNCs, respectively [12, 116]. Induction of miR-532-5p by hypoxia results in ANG1 downregulation and pericyte dysfunction. Whereas, in MNCs, induction of miR-210 by hypoxia leads to ephrin A3 downregulation eventually enhancing the MNC proangiogenic potential. Notably, ex vivo transfection of MNCs with miR-210 overexpressing vectors resulted in enhanced angiogenesis stimulation following transplantation in a mouse model of limb ischemia [12].

MiRNAs were also identified as important regulators of adipose tissue functions and may link inflammation and insulin resistance, as reviewed in [117]. Interestingly, some of these adipo-miRNAs are also expressed by vascular cells and implicated in T2D and ageing-associated vasculopaties [118].

These observations collectively point to miRNAs as pivotal players in the onset and progression of T2D and CLI. Consequently, miRNAs represent viable targets for the development of therapeutic strategies including ex vivo expression level modification in candidate cell products. Table 2 provides a list of deregulated miRNAs in T2D- related ED/CLI.

**Table 2:** MiRNAs differently expressed in patients with T2D affected by CLI or ED. EPCs, endothelial progenitor cells; *HUVECs*, human umbilical vein-derived endothelial cells; *miRNA*, microRNA; *MNCs*, mononuclear cells; *PB*, peripheral blood

miRNAs affected by ED/CLI in T2D	Up/downregulation	Sample type
miR-15a	Up	PB-EPCs, serum [115••]
miR-16-1	Up	PB-EPCs, serum [115••]
miR-130a	Down	EPCs [119]
miR-126	Down	EPCs [111]
miR-126-3p	Down	Plasma [120]
miR-21	Down	EPCs [82•]
miR-21-5p	Up	Plasma [120]
miR-200b-3p	Up	Plasma [121]
miR-2115-3p	Up	Plasma [121]
miR431-5p	Up	Plasma [121]
miR-486-5p	Up	Plasma [121]
miR-210-3p	Up	Plasma [121]
miR-1264	Up	Plasma [121]
miR323b-5p	Up	Plasma [121]
miR-5579-3p	Down	Plasma [121]
miR-665	Down	Plasma [121]
miR-4285	Down	Plasma [121]
miR-500a-3p	Down	Plasma [121]
miR-4739	Up	Whole blood [122]
miR-342-3p	Up	PB EPCs [123]
miR-29	Down	Arteriole biopsies [124]
miR-149-5p	Down	HUVECs [125]
miR-4463	Up	Femoral arteries, HUVECs [126]
miR-423	Down	Plasma [127]
miR-199a-3p	Down	PB [128]
miR-93	Up	Plasma [129]
miR-23c	Up	PB-MNC, biopsies [130]
miR-92a	Up	PB [131]

### Long Noncoding RNAs in Diabetes-Related Vascular Disease: Implications for Cell Therapy

LncRNAs represent a class of transcribed RNA molecules that are longer than 200 nucleotides and yet do not encode proteins. Several lncRNAs participate in the regulation of stem cell properties including their potential for self-renewal and differentiation, which help in maintenance of normal cell and tissue homeostasis. We have recently reported a T2D-dependent mechanism involving miR-21 downregulation CD34^+^ MNCs and induction of its inhibitory target PDCD4, a tumor suppressor protein involved in programmed cell death [82•]. We also found that several lncRNAs that act as sponges for miR-21 were overexpressed in CD34^+^ MNCs and PB of patients with T2D. We proposed that this altered paracrine signalling may convey antiangiogenic and proapoptotic features from CD34^+^ cells to the endothelium [82•]. The study has important implications for cell therapy, calling for caution in using CD34^+^ cells that carry a proapoptotic and antiangiogenic molecular signature, e.g., low miR-21/high PDCD4.

Several lncRNAs are gathering attention as theragnostic tools in vascular complications of T2D as reviewed in [132]. An example can be found in MALAT-1, which is one of the most studied lncRNAs expressed in ECs, where it regulates hyperglycaemia-induced inflammatory process [133]. Moreover, its knockdown was associated to an amelioration of ED [134]. Interestingly, MALAT-1 is also upregulated in CD34^+^ cells from patients with T2D, suggesting a general implication in vascular dysfunction and cell-related repair processes [82•]. Another lncRNA, MEG-3, was associated with a protective effect towards high glucose (HG)-treated HUVECs [135].

Recent evidence suggests a role for circRNAs in diabetes-related epigenetic regulation. CircRNAs represent a large class of stable RNAs produced by circularization of specific exons. In vivo, they are generated by the spliceosome via backsplicing and can modulate gene expression through sequestration of RNA-binding proteins, transcriptional regulation, or sequestration of their target miRNAs [136–138]. cPWWP2A is a circRNA overexpressed by retinal pericytes of streptozotocin-induced diabetic mice. It can be delivered to ECs through EVs-mediated paracrine signalling, resulting in miR-579 sequestration and consecutive overexpression of ANG1, occludin, and sirtuin 1, thereby providing a protective effect [139]. Likewise, HG condition triggers another protective mechanism involving the circRNA-0054633-mediated downregulation of miR-218, eventually contrasting ED [140]. On the other hand, overexpression of circHIPK3 has a deleterious effect in diabetic retinopathy through sponging of miR-30a-3p [141]. Likewise, circRNA-ZNF609 silencing was shown to ameliorate ED in a diabetic mice model [142]. Another study showed that culturing HUVECs under HG conditions activates circ-0068087, a sponge circRNA responsible for the upregulation of NF-κB-mediated inflammation through the downregulation of its target miR-197 [143].

Recent sequencing studies have advanced the understanding of diversity in non-coding RNAs due to T2D. In a high throughput RNA-sequencing study, the comparison between blood samples from three just-diagnosed patients with T2D and three healthy patients unravelled a multitude of differentially expressed miRNAs, lncRNAs, circRNAs as well as mRNAs [144]. Furthermore, another RNA-sequencing study comparing patients with diabetes with and without diabetic foot complications showed that 632 mRNAs and 33 circRNAs were differently expressed in the sera of the two groups [145].

Taken together, these results provide a plethora of potential molecular targets to improve the functionality of autologous cell therapy in the treatment of NO-CLI.

## Conclusions

The quest for an alternative treatment to surgical amputation for NO-CLI patients relentlessly strives on, as reported in this short review, accompanied by the steadily expanding account of the molecular networks embedded in the pathological milieu. Several studies reported here highlighted the importance of stem and progenitor cells as well as ncRNAs in such context. The constantly growing acknowledgement of the molecular connections between CLI and T2D suggests novel therapeutic approaches will rise soon. We can therefore conclude that future trends in CLI cell therapy will keep on pursuing the refinement of the ideal strategy to achieve tissue reperfusion, TcPO2 increase, pain at rest reduction, as well as improvement of painless walking distance, AFS rate, and mortality rate. Additionally, miRNAs emerge as essential therapeutic and diagnostic targets as they are broadly involved in the mechanisms of neovascularization. The wide range of recently discovered angiogenic miRNAs associated with every known aspect of CLI pathophysiology is likely to provide a solid base to boost the sprouting of innovative strategies and refine current cell therapy techniques soon. The definition of reliable predictors of responsiveness to a given treatment should also enhance the efficacy of future cell therapy trials [146]. Finally, evidence reported in meta-analyses indicate that large, double-blind, placebo-controlled, randomized trials with long-term follow-up are mandatory to firmly establish cell therapy as an acceptable treatment of CLI.

## Abbreviations

ABI: Ankle-brachial index
AFS: Amputation-free survival
ANG: Angiotensin
ANG1: Angiopoietin 1
BM: Bone marrow
BM-MNCs: Bone marrow-derived mononuclear cells
CCL2: Chemokine C-C motif ligand 2
circRNAs: Circular RNAs
CLI: Critical limb ischaemia
cBMA: Concentrated bone marrow aspirate
CXCL12: C-X-C motif chemokine 12
CXCR4: C-X-C chemokine receptor type 4
ECs: Endothelial cells
ED: Endothelial dysfunction
eNOS: Endothelial nitric oxide synthase
EPCs: Endothelial progenitor cells
ETC: Electron transfer chain
EVs: Endothelial vescicles
FGF: Fibroblast growth factor
GFs: Growth factors
G-CSF: Granulocyte colony stimulating factor
HGF: Hepatocyte growth factor
hiPSCs: Human-induced-pluripotent stem cells
HPSCs: Haematopoietic progenitor stem cells
HSCs: Haematopoietic stem cells
IGF-1: Insulin growth factor-1
IRS-1: Insulin receptor substrate-1
MAMs: Mitochondria-associated membranes
MHC: Mayor histocompatibility complex
mTOR: Mammalian target of rapamycin
miRNAs: Micro RNAs
MNCs: Mononuclear cells
MSCs: Mesenchymal stromal cells
lncRNA: Long non-coding RNA
ncRNA: Non-coding RNA
NO-CLI: No option CLI
PB: Periphral blood
PB-MNCs: Peripheral blood-derived mononuclear cells
PDCD4: ProgrammeD cell death 4
PDGF: Platelet-derived growth factor
PVD: Peripheral vascular disease
ROS: Reactive oxidative species
SASP: Senescent activated secretory phenotype
T2D: Type 2 diabetes
TcPO2: Transcutaneous oxygen pressure
ULK1: Unc-51 like autophagy activating kinase 1
VEGF: Vascular endothelial growth factor
VSMC: Vascular smooth muscle cells
